# Tailoring the electronic and magnetic properties of monolayer SnO by B, C, N, O and F adatoms

**DOI:** 10.1038/srep44568

**Published:** 2017-03-14

**Authors:** Junguang Tao, Lixiu Guan

**Affiliations:** 1 Key Lab. for New Type of Functional Materials in Hebei Province, School of Materials Science and Engineering, Hebei University of Technology, Tianjin 300130, China; 2 School of Science, Hebei University of Technology, Tianjin 300401, China

## Abstract

Recently, SnO has attracted more and more attention, because it is a bipolar electronic material holding great potential in the design of *p-n* junction. In this paper, we examine the effect of extrinsic point defects on modifying the electronic and magnetic properties of SnO using density functionals theory (DFT). The surface adatoms considered are B, C, N, O and F with a [He] core electronic configuration. All adatoms are found energetically stable. B, C, N and F adatoms will modify the band gap and introduce band gap states. In addition, our calculations show that N, B and F can introduce stable local magnetic moment to the lattice. Our results, therefore, offer a possible route to tailor the electronic and magnetic properties of SnO by surface functionalization, which will be helpful to experimentalists in improving the performance of SnO-based electronic devices and opening new avenue for its spintronics applications.

Since the discovery of graphene, two-dimensional (2D) materials with atomic thickness have drawn increased attentions in recent years that hold promise for developing next-generation high-performance electronics, optoelectronics and spintronics^1^,^2^,^3^,^4^,^5^,^6^,^7^,^8^. Many exotic physical and chemical phenomena emerge with reducing dimensions owing to the change of electronic behaviors governed by quantum confinement effects within the 2D layer. Although excellent electrical and thermal transport properties make graphene a promising candidate for applications in transparent conductor and high mobility devices, its zero bandgap nature limits its technological applications in digital electronic devices, which inspires researches for materials with finite bandgap, such as transition metal dichalcogenides (TMDCs), (MX_2_, M a transition metal atom, such as Mo, W, Re, Pt, Sn etc. and X a chalcogen atom, such S, Se, or Te)^9^,^10^,^11^ and black phosphorus^12^ (BP) that were recently extensively studied. More recently, layered tin (II) oxide (SnO) was found to show excellent semiconducting performances in which bipolar conductivity can be easily realized^13^,^14^,^15^,^16^. It is a rare example of layered oxide semiconductors that holds promise for a wide variety of technological applications^14^,^17^,^18^, which makes it quickly become the subject of significant theoretical and experimental investigations^1^,^13^,^14^,^18^,^19^. Compared to other TMDC-based layered structure, oxide-based materials are expected to be more stable in air since they are reluctant to oxidation.

Bulk SnO has a tetragonal unit cell (litharge crystal structure with space group: *P4/nmm*) with lattice constants of *a = b* = 3.8 Å and *c* = 4.84 Å^20^,^21^. A lone pair model was suggested for the electronic interaction of SnO^22^, in which the lone pair states result from the crystal structure of SnO. In SnO, each Sn atom loses two 5*p* electrons to adjacent O atoms leaving an electronic configuration of 4*d*^*10*^5 *s*^*2*^5*p*^*0*^ in which the two Sn 5*s* electrons constitute a lone pair pointing towards the interlayer spacing. The inter-layer lone-pair interaction was found to be crucial for understanding the electronic structure of SnO^14^. In analogy with other TMDCs-based layered structure, Sn–O–Sn slabs in SnO are stacked along [001] direction with weak dipole-dipole van der Waals interlayer interaction^1^,^14^,^15^. In its monolayer counterpart, the lack of dipole-dipole lone pair interaction shows great impact on the electronic structure and widens the band gap which bring additional potential applications. To design a SnO-based material with required electronic structure, it is essential to explore the relationship between the engineering methods and their effects on the electronic and magnetic modifications. The unique lone pair electronic states around Sn atoms could also be modified by adatoms which needs to be understood.

In order to explore the potential applications of 2D materials, many strategies could be used to tune their electronic and magnetic properties, such as strain^15^, defects and substitutional doping^23^. It is well-known that the exfoliation or growth processes can introduce defects and impurities in 2D materials which can dramatically alter their electronic, thermal and mechanical properties. Vice versa, a deliberate introduction of defects can be a feasible approach to modify the properties of the pristine materials. For instance, vacancies and Stone-Wales (SW) defects introduced in graphene by ion or electron irradiation brought new functionalities for graphene-based devices^24^,^25^. Defects in MoS_2_ formed during the growth process also play significant roles on their electronic behaviors as well as device performance^26^,^27^,^28^. Besides intrinsic defects, extrinsic defects such as adatoms are also shown to be important for 2D materials based devices with dedicated properties^29^. For real applications, modifications and engineering are generally applied to enhance the materials/devices functionalities. Especially for new materials, great efforts are needed to explore its function enhancement in all manner of possibilities. Adsorption of foreign atoms is another effective and promising strategy for tuning the electronic and magnetic properties of 2D materials^23^,^30^. Considering that the scientific investigations on the properties of SnO has just started^14^,^15^,^16^,^18^,^19^,^31^,^32^,^33^,^34^, the role of extrinsic point-defects and the effects of adatoms on SnO need to be explored to widen the range of its applications.

In this work, we systematically investigate the stable crystal structures and electronic and magnetic properties of nonmetal atoms (B, C, N, O, and F) adsorbed SnO monolayers. We will calculate their geometric structure and electronic properties to gain insights in the adsorption mechanism. Compared with the magnetic moment from *d*-electrons of transitional-metal atoms, the magnetism from *sp* electrons of nonmetal elements could have stronger long-range exchange coupling interactions and avoid cluster formation of magnetic ions^35^. We will show that the electronic behavior of C and O is totally different from that of B, N and F adatoms, and the adsorption mechanism is dominated by the electronegativity of the adatom.

## Results and Discussion

### Band structure property

**Figure 1:**
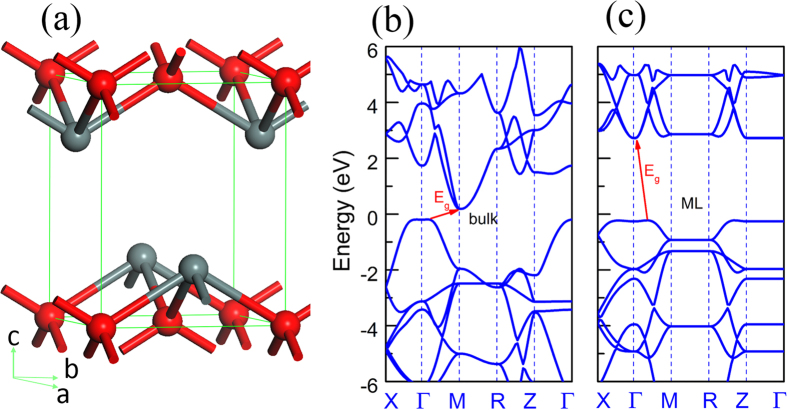
(**a**) Ball-and-stick model for SnO unit cell. The band structures of SnO bulk and monolayer are given in (**b**) and (**c**). The energy zero is set to the top of valence band.

SnO possesses a layered structure with each Sn atom bonded with four adjacent O atoms and vice versa, see the ball-and-stick model in Fig. 1(a). In Fig. 1, the band structure at the valence band maximum (VBM) and conduction band minimum (CBM) for both bulk and monolayer (ML) SnO are given. It is found that both bulk and ML SnO are indirect band gap semiconductor, in which VBM is situated among Γ-M line. However, the CBM is located at M point for bulk and shifted to Γ point for ML which is in excellent agreement with previous theoretical reports^13^,^14^,^15^. The involvement of spin-orbital coupling (SOC) is found to have no effect on the band structure, *i.e.* no band splitting at CBM and VBM (Fig. S1). The evolution of band structure implies indirect-direct-indirect band bap conversion may occur during the layer-thinning process, which is in strong contrast to other 2D layered materials, such as TMDCs. For the bulk SnO, the band gap is 0.38 eV while it increases dramatically to 2.94 eV for ML SnO at GGA level, see Fig. 1(b,c). The huge change of the band gap highlights the importance of interlay coupling which affects significantly the lone pair states that could also be modified by adsorption of foreign atoms. As is well known, the band gap is underestimated by the standard DFT calculation due to the poor description of the correlation interaction between electrons. To overcome this drawback, GGA+U and HSE06 methods were used to correct this. The results show that the correction of band gap do not have noticeable effect on the band structure behaviour (Figs S2–S3). Although interaction in the [001] direction is governed by weak van der Waals, the calculated band structure for bulk SnO [see Fig. 1(b)] exhibits significant band dispersion along the Γ-Z direction at VBM. However, Fig. 1(c) shows that the band dispersion along *z* directions become flat for ML SnO which implies the band dispersion is originated from the strong inter-layer interaction. This is a very different behavior from the transition-metal dichalcogenides^9^. The orbital contributions to the electronic structure of pristine bulk SnO is obtained from partial density of states (PDOSs) which shows the VBM is derived from the in-layer hybridization of Sn 5*sp* orbitals and the O 2*p* orbitals consistent with lone pair model^22^, while the CBM is contributed primarily from the hybridization of Sn 5*p* and O 2*s* antibonding orbitals and associated with the interlayer Sn^2+^–Sn^2+^ bonds. Since the band gap evolution is dominated by the orbitals at CBM, it is the interlayer lone pair interaction between Sn atoms that dominates the electronic structure and the band gap of SnO. Our calculated results are in excellent agreement with the previous theoretical reports^14^,^36^.

### Geometry stabilization

**Figure 2:**
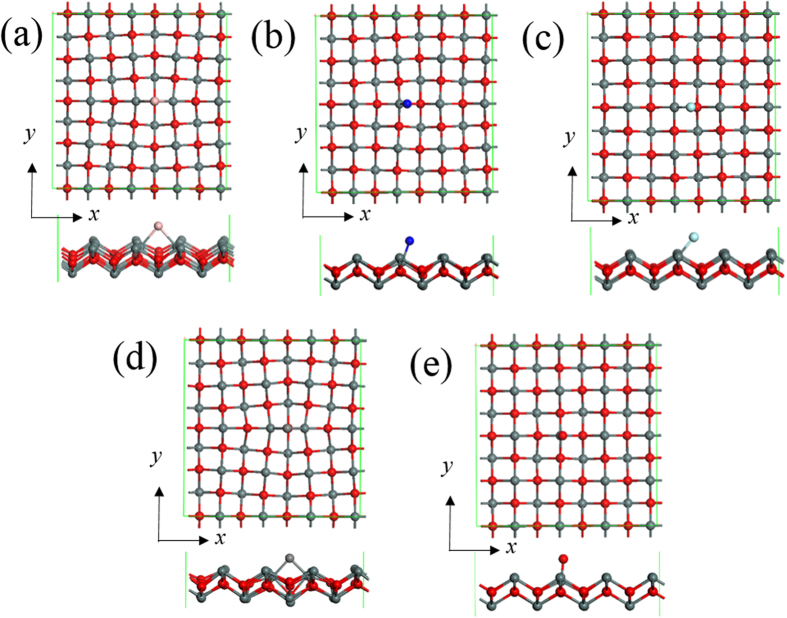
Top view and side view of relaxed geometric structure for B- (**a**), N- (**b**), F- (**c**), C- (**d**) and O-adsorbed (**e**) SnO monolayer. The red atoms are O and the gray atoms are Sn.

In SnO, each Sn atom shares its two 5*p* electrons with the neighboring O atoms while the remaining 5*s* electrons do not participate in the bonding process and constitute a lone pair. As mentioned above, the band structure of SnO is determined by the lone pair interaction, the above model gives a strategy for the electronic structure design by adatoms which bond with Sn atoms via the lone pair electrons. To do so, the adsorption of a series of adatoms of B, C, N, O and F were studied, which provides an interesting variation in the number of valence electrons with a [He] core and electronegativity. After adsorption, the geometric structure was changed as shown in Fig. 2(a–e). In the equilibrium configurations, the preferred binding sites are between two surface Sn atoms. B, C, N and F adatoms prefer the bridge site whereas O adatom prefers the top site. On the other hand, B and C adatoms appear to form bonds with two native Sn atoms at the surface in their equilibrium configurations. It is noted from Fig. 2 that the surface reconstruction is pronounced in B- and C-adsorbed SnO monolayers. The native Sn atoms along *x* direction are attracted toward the adatoms while repelled along *y* direction resulting in lattice distortions with a positive Poisson’s ratio. It is noticed that the adsorption of additional O atoms has little effect on the lattice distortion, see Fig. 2(e). SnO bond length in the pristine 2D lattice is 2.257 Å. For all adatoms expect B, the Sn-X (X = C, N, O and F) distance is shorter than that of pristine Sn-O bond suggesting strong bonding. The binding energy (*E*_*B*_) of the surface adatom is defined as


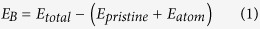


where *E*_*total*_ is the total energy of SnO with surface adatom, *E*_*pristine*_ is the energy of pristine SnO, *E*_*atom*_ is the energy of a single adatom calculated using a 10 × 10 × 10 Å^3^ unit cell. According to the definition of binding energy, the negative value of *E*_*B*_ implies that the adsorption process is exothermic, and the larger is the absolute value of *E*_*B*_, the stronger is the interaction between the adatom and SnO monolayer. As shown in Table 1, the calculated binding energies of B, C, N, O, and F adatoms on SnO are −1.765, −3.495, −4.243, −5.419, and −3.820 eV, respectively, which are stronger than they adsorbed on graphene^37^,^38^,^39^. The binding strength order is also in consistent with their bonding length. Since B, C, N, O and F atoms are more electronegative than the host Sn atoms, these adatoms tend to attract electrons from the lattice Sn atoms.

### Magnetic property

Having established the stability of X (X = B, C, N, O and F) defects on 2D SnO, we start to look at the changes in the electronic and magnetic properties of the host SnO by an X atom. In Table 1, it can be found that C and O atoms do not introduce magnetic moments to the system as B, N and F do. The magnetic energy (*ΔE*_*m*_), which represents the energy gained from the spin polarization, is calculated as:


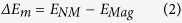


for the decorated SnO monolayer, where *E*_*NM*_ and *E*_*Mag*_ are the total energy for non-magnetic and magnetic system, respectively. The magnetic energies of O-, C-, B-, N- and F-adsorbed SnO monolayers are 1.3, 14.3, 280.8, 471.7 and 134.8 meV, respectively. The obtained *ΔE*_*m*_ for B-, N- and F-adsorbed SnO are higher than thermal fluctuation of the surroundings. Therefore, the B-, N- and F-adsorbed SnO monolayers exhibit stable magnetic ground states with total magnetic moment of 0.41, 0.63 and 0.79 *μ*_*B*_ per adsorption, respectively. The O- and C-adsorbed SnO monolayers are nonmagnetic because their magnetic moment are zero.

**Figure 3:**
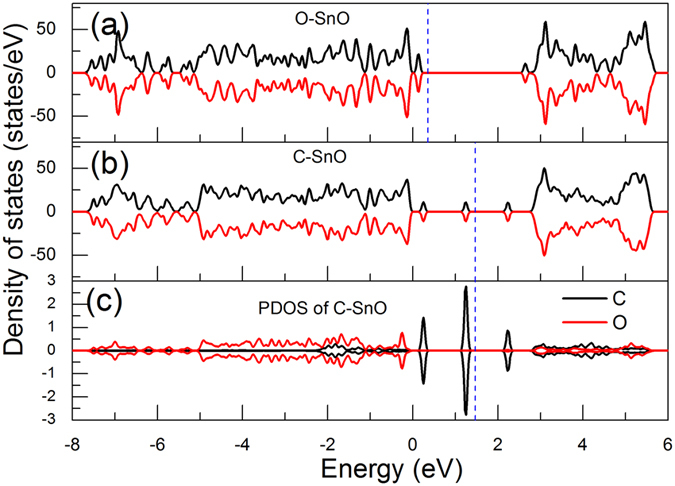
Spin polarized density of state (DOS) spectra for O- (**a**) and C-adsorbed (**b**) SnO monolayer. For C-SnO system, the *p* orbitals of C adatom and lattice O atom is given in (**c**). The energy zero is set to the top of valence band. Blue dashed lines correspond to the Fermi levels.

In order to explore the mechanism of the difference in forming magnetic moment for different adatoms, we first present the spin-resolved density of states (DOS) for O and C adsorbed cases in Fig. 3. The adsorption gives rise to highly localized states within the band gap, as shown in the DOS plots in Fig. 3. From the spin resolved DOS spectra, the additional states introduced by O atom sit just on top of VBM and below CBM while C adsorption introduces three deeper mid-gap states. However, in both cases, the up-spin and down-spin are symmetrical which cancel each other resulting in nonmagnetic ground states. Although the O adsorption has a small effect on the electronic properties of ML SnO within the gap, [Fig. 3(a)], the new states it introduced reduce the band gap by 0.44 eV. In comparison with the O adsorption, the C adatom brings a deeper *n*-type and *p*-type mid-gap states with negligible effect on the matrix band gap [see Fig. 3(b,c)].

**Figure 4:**
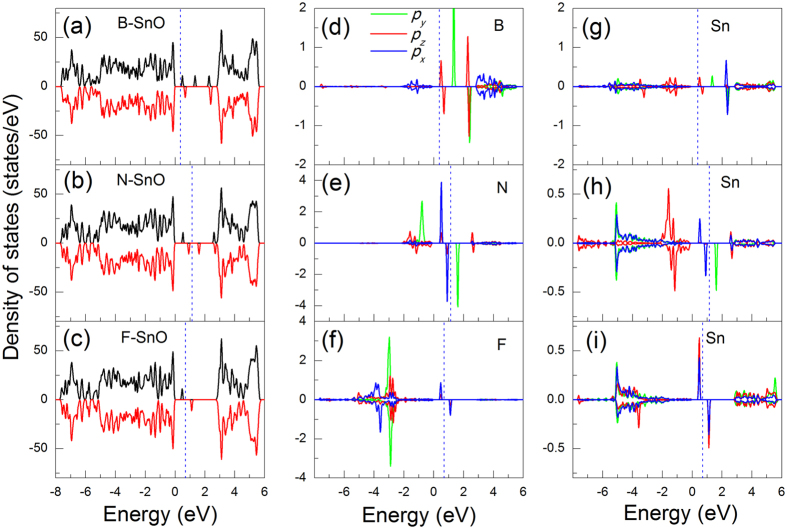
Spin polarized total density of state (DOS) spectra for B- (**a**), N- (**b**) and F-adsorbed (**c**) SnO monolayer. (**d**,**e**) and (**f**) are the PDOS of the corresponding adatoms for B, N, and F. (**g**,**h**) and (**i**) are the PDOS of the corresponding Sn adatoms that the foreign adatoms adsorbed onto. The energy zero is set to the top of valence band. In (**d**–**i**), the green, red, and blue solid curves are for *p*_*y*_*, p*_*z*_ and *p*_*x*_ orbitals, respectively. Blue dashed lines correspond to the Fermi levels.

**Figure 5:**
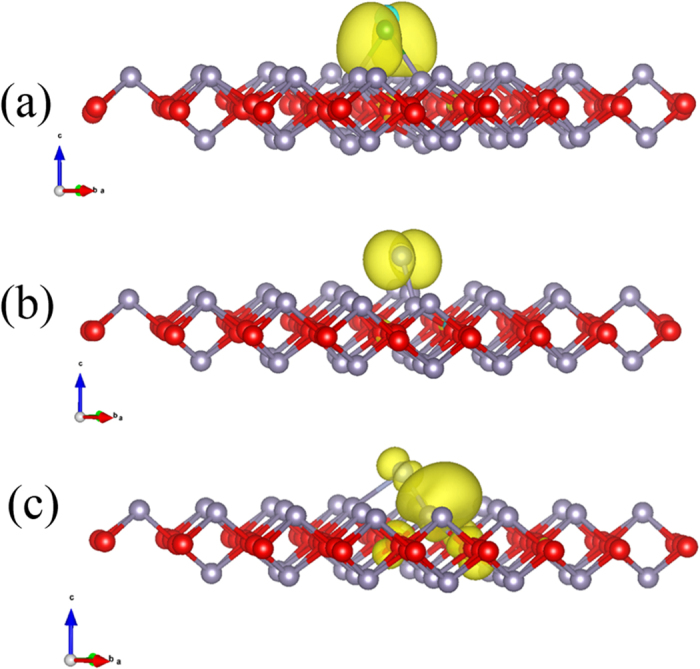
Spin charge density for B- (**a**), N- (**b**) and F-adsorbed (**c**) SnO monolayer. The isovalues are set at 0.002 e/Å^3^.

Since all B, N and F adsorptions bring in magnetic moments to the system, it is neccesarry to explore their nature. Figure 4 displays spin and atom resolved DOS of the adsorbed systems. All B, N, and F atoms induce mid-gap states in the band gap of pristine SnO. Different from B and F, adsorption of N introduces a state just below CBM thus reduces the band gap by 0.24 eV. Adsorption of B, N, or F also results in the spin polarized DOS [Fig. 4(a–c)] leading to noticeable magnetic moments less than 1 μ_B_. The spin polarized charge density is found to be mainly localized on the adatoms and nearby Sn atoms, see Figs 4(d–f) and 5. In B adsorption case, all the mid-gap states are originated from B 2*p*_*y*_ and *2p*_*z*_ orbitals, while its 2*p*_*x*_ orbitals are mainly spread within conduction bands, see Fig. 4(d). In addition, the spin splitting for B 2*p*_*z*_ orbitals is less than that of 2*p*_*y*_ orbitals, which is in agreement with the spin charge density distribution as illustrated in Fig. 5(a). Although the adsorption site of C atom is similar to that of B atom, C atom has one more valence electron than B atom, which could possibly pair up with the electrons associated with Sn atom resulting in zero net magnetic moment. On the other hand, the mid-gap states are originated from 2*p*_*y*_, *2p*_*x*_ and *2p*_*z*_ orbitals of N atom [Fig. 4(e)] and 2*p*_*x*_ orbitals of F atom [Fig. 4(f)], respectively. In all these three cases, the spin splitting is mainly originated from the in-plane *p* orbitals, i.e. *p*_*x*_ and/or *p*_*y*_ orbitals and the hybridization of adatoms is formed with Sn 5*p* orbitals [see Fig. 4(g–i)]. The strong hybridization between the adatoms and matrix Sn atom demonstrates the formation of covalent bond.

We note that all B, N and F atoms have odd number of 2*p* electrons while C and O has even number of 2*p* electrons. Therefore, the introduced magnetic moments are originated from the unpaired electrons for B, N and F adsorption cases. All B, N and F adatoms will attract the electron from the lone pair of Sn leaving an unpaired electron on the 2*p* orbitals of the native Sn atom, which contributes to the calculated magnetic moment. For example, B has *s*^2^*p*^1^ electron configuration attracting the lone pair of Sn atom, possibly forming Sn-B bond with a larger bond length of 2.518 Å. N has *s*^2^*p*^3^ configuration and will form bond with two native Sn atoms. F has *s*^2^*p*^5^ valence electron configuration and will attract one electron of the lone pair forming bond with one native Sn atom. Their bonding strength can be quantified by the binding energies as given in Table 1 which is in line with their bonding lengths. In addition, the total magnetic moments they introduced monotonically increase for B-, N-, and F-SnO which is consistent with trend of their electronegativity values.

The spin charge density of B, N and F adsorbed cases is presented in Fig. 5. The yellow region represents accumulation of spin density and the isovalue is 0.002 e/Å^3^. It is shown that the spatial extensions of spin density changes with atomic numbers: the higher the atomic number of the adsorbate, the less spin density distribution on the adatoms and more on matrix Sn atoms. This suggests that the magnetic moment is mainly contributed from the adatoms and their proximate Sn atoms.

### Summary and conclusion

In summary, we present a systematic study on the stable configurations, electronic structures, and magnetic properties of B-, C-, N-, O- and F-adsorbed SnO monolayers. It is found that all these adatoms can be chemically adsorbed on the surface of SnO substrate. The electronic structure of SnO monolayer can be widely tuned by the adsorbed adatoms. The B-, N- and F-adsorbed SnO monolayers exhibit magnetic ground states, and the contributions of B or N to the total magnetic moments are weaker than that of F. Our studies suggest that nonmetal atoms adsorption is an effective approach to tune the electronic and magnetic properties of SnO monolayer, which opens an alternative way for future optoelectronic and spintronic applications.

### Calculations details

Our calculations are performed using the Vienna Ab-initio Simulation Package (VASP)^40^,^41^,^42^. The valence and core interactions are treated by the projected augmented wave (PAW) method^43^ and the exchange correlation energy was described by the generalized gradient approximation (GGA)^44^. The Kohn-Sham orbitals were expanded in a plane-wave basis with a cutoff energy of 500 eV and the exchange-correlation functional is treated by Perdew-Burke-Ernzerhof form generalized gradient approximation (GGA-PBE)^44^. The Brillouin zone (BZ) was sampled by 3 × 3 × 1 Monkhorst-Pack *k*-point grids. The convergence threshold for self-consistent field (SCF) energy is set at 10^−6^ eV, and all the atomic positions are fully optimized until the Hellman-Feynman forces are smaller than 0.01 eV/Å.

## Additional Information

**How to cite this article:** Tao, J. and Guan, L. Tailoring the electronic and magnetic properties of monolayer SnO by B, C, N, O and F adatoms. *Sci. Rep.* **7**, 44568; doi: 10.1038/srep44568 (2017).

**Publisher's note:** Springer Nature remains neutral with regard to jurisdictional claims in published maps and institutional affiliations.

## Supplementary Material

Supplementary Information

## Figures and Tables

**Table 1:** Adsorption of adatoms with a [He] core on SnO monolayer. The bonding length (*R*_Sn-adatom_ Å), binding energy (*E*_B_, eV), magnetic stabilization energy (∆*E*_M_, meV), total magnetic moment (μ_B_), the magnetic moment of adatom (μ_B_) and their surrounding Sn atoms (μ_B_).

Defects	*R*_Sn-adatom_ (Å)	*E*_B_ (eV)	∆*E*_M_ (meV)	Total magnetic moment (μ_B_)	Magnetic moment of adatom (μ_B_)	Magnetic moment of surrounding Sn (μ_B_)
Nonmagnetic defects						
O	1.864	−5.419	1.3	0.0	0.0	0.0
C	2.160	−3.495	14.3	0.0	0.0	0.0
Magnetic defects						
N	2.006	−4.243	471.7	0.63	0.61	0.01
B	2.518	−1.765	280.8	0.41	0.26	0.09
F	2.080	−3.820	134.8	0.79	0.12	0.34
