# Supplementary material for: Tailoring the electronic and magnetic properties of monolayer SnO by B, C, N, O and F adatoms
Source: Sci Rep. 2017 Mar 14;7:44568. doi: 10.1038/srep44568 (PMC5349556; doi:10.1038/srep44568)
Supplement: Supplementary Information [file srep44568-s1.pdf]

Supplementary information for

Tailoring the electronic and magnetic properties of monolayer SnO by B, C, N, O and F adatoms

Junguang Tao,[1] Lixiu Guan,[2,]*

[1]Key Lab. for New Type of Functional Materials in Hebei Province, School of Materials Science and Engineering, Hebei University of Technology, Tianjin 300130, China

[2]School of Science, Hebei University of Technology, Tianjin 300401, China

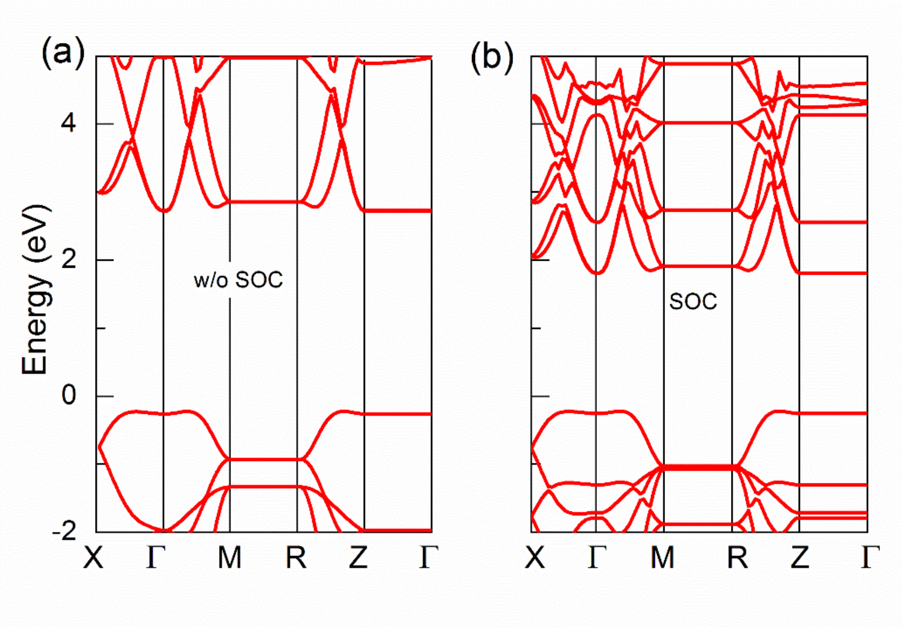

Fig. S1. (Colour online) Band structure of ML SnO without (a) and with (b) spin-orbital coupling (SOC).

As is well known, the band gap is underestimated by the standard DFT calculation due to the poor description of correlation interaction between electrons. To overcome

this drawback, GGA+U (with Dudarev method) and HSE06 methods were used to test the effect of electron correlation interaction on their electronic structures. As shown in Fig. S2, both GGA+U and HSE06 treatment will increase the band gap of SnO bulk. Other than the band gap opening, no noticeable changes can be observed for the band structures with both GGA+U and HSE06 methods. Since HSE06 method is more time consuming, we performed further verification based on GGA+U method. Surprisingly, the GGA+U does not modify the band gap of the SnO monolayer (ML), see Fig. S3 for U = 3, 5 and 7 eV. Moreover, the density of states at VBM and CBM are almost identical as that of U = 0. In addition, a test run for U = 5 eV was performed for the F-SnO case. The DOS spectra are almost identical as that of U = 0 eV, see Fig. S4 and Fig. 4. The conclusions to be drawn are as same as U = 0 eV. For instance, the magnetic moment of the F-SnO system at U = 5 eV is only increased to 0.80 μ_B as compared to 0.79 μ_B for U=0. Therefore, the plus-U method does not affect our conclusions.

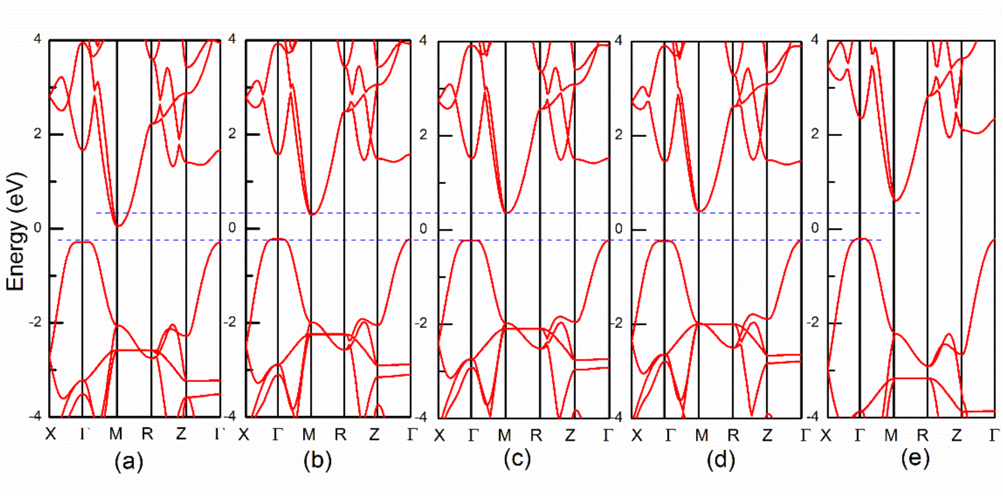

Fig. S2. (Colour online) Band structure of SnO bulk for different U values and HSE06 method. (a) U = 0 eV; (b) U = 3 eV; (c) U = 5 eV; (d) U = 7 eV; and (e) HSE06. The horizontal dashed blue line are drawn to guide the eyes.

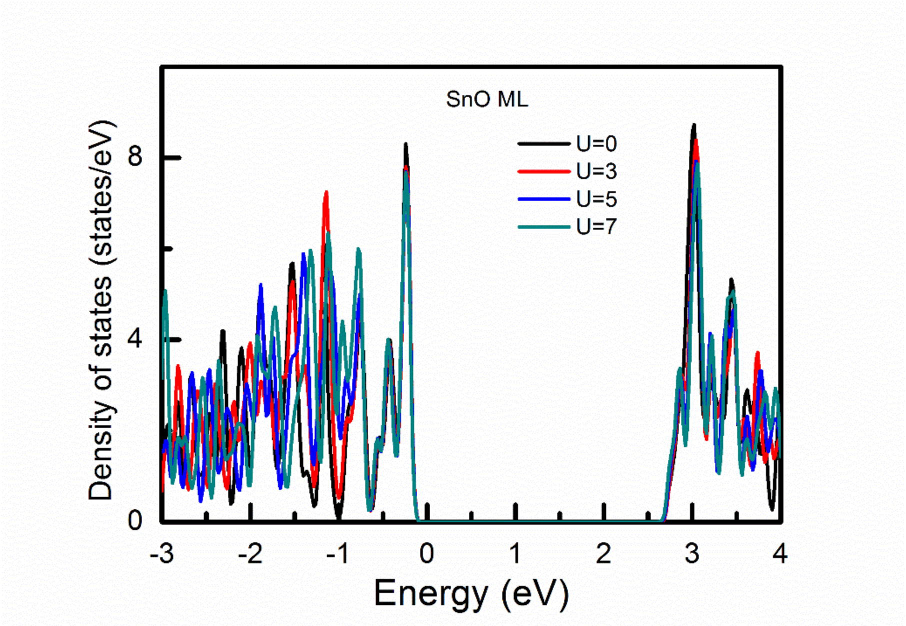

Fig. S3. (Colour online) Density of states for ML SnO with different U values.

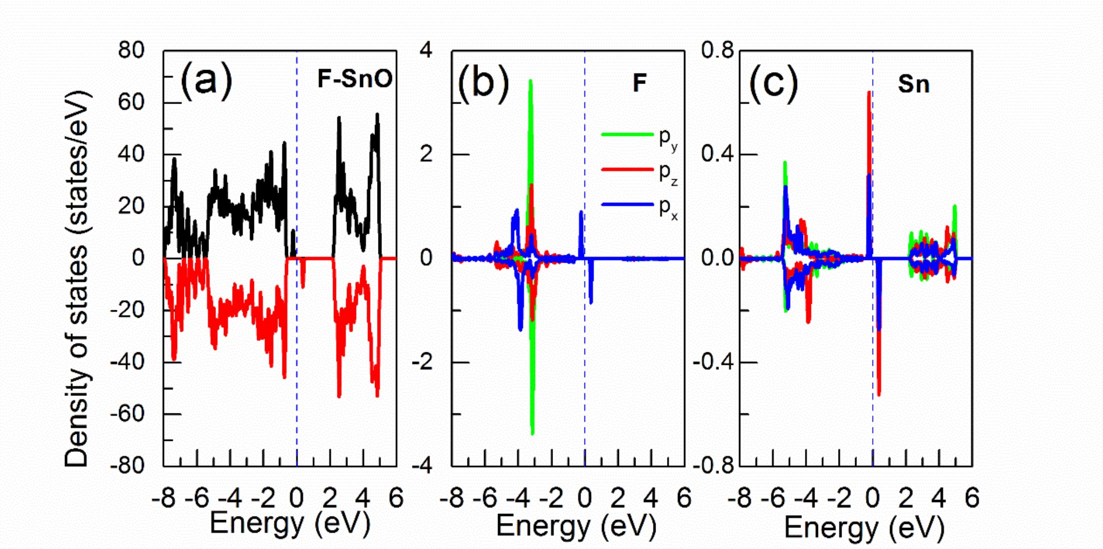

Fig. S4. (Colour online) Spin polarized total density of state (DOS) spectra for F-SnO monolayer with GGA+U (U = 5 eV) method. (b) is the PDOS of the corresponding F adatom. (c) is the PDOS of corresponding Sn adatoms that the F adatom adsorbed onto. Fermi level is set to energy zero and indicated by the blue dashed lines.
